# Total Laboratory Automation and Matrix-Assisted Laser Desorption Ionization–Time of Flight Mass Spectrometry Improve Turnaround Times in the Clinical Microbiology Laboratory: a Retrospective Analysis

**DOI:** 10.1128/JCM.01242-17

**Published:** 2017-12-26

**Authors:** Talent Theparee, Sanchita Das, Richard B. Thomson

**Affiliations:** a Department of Pathology and Laboratory Medicine, NorthShore University HealthSystem, Evanston, Illinois, USA; Mayo Clinic

**Keywords:** MALDI-TOF MS, clinical microbiology, total laboratory automation, turnaround time

## Abstract

Technological advances have changed the practice of clinical microbiology. We implemented Bruker matrix-assisted laser desorption ionization–time of flight mass spectrometry (MALDI-TOF MS) and BD Kiestra total laboratory automation (TLA) 4 and 3 years ago, respectively. To assess the impact of these new technologies, we compared turnaround times (TATs) for positive and negative urine cultures before and after implementation. In comparison I, TATs for 61,157 urine cultures were extracted for two periods corresponding to pre-TLA and post-TLA, both using MALDI-TOF MS for organism identification. In comparison II, time to organism identification (ID) and antimicrobial susceptibility (AST) reports were calculated for 5,402 positive culture reports representing four different periods: (i) manual plating and conventional biochemical identification (CONV), (ii) manual plating and MALDI-TOF MS identification (MALDI), (iii) MALDI-TOF MS identification and early phase implementation of TLA (TLA1), and (iv) MALDI-TOF MS identification and late phase implementation of TLA (TLA2). By the comparison I results, median pre- and post-TLA TATs to organism IDs (18.5 to 16.9 h), AST results (41.8 to 40.8 h), and preliminary results for negative cultures (17.7 to 13.6 h), including interquartile ranges for all comparisons, were significantly decreased post-TLA (*P* < 0.001). By the comparison II results, MALDI significantly improved TAT to organism ID compared to CONV (21.3 to 18 h). TLA further improved overall TAT to ID (18 to 16.5 h) and AST (42.3 to 40.7 h) results compared to MALDI (*P* < 0.001). In summary, TLA significantly improved TAT to organism ID, AST report, and preliminary negative results. MALDI-TOF MS significantly improved TAT for organism ID. Use of MALDI-TOF MS and TLA individually and together results in significant decreases in microbiology report TATs.

## INTRODUCTION

Rapid reporting of microbiology culture results is of utmost importance in the management of infectious diseases and is essential for patient care. Although a relatively new concept in clinical microbiology, total laboratory automation (TLA) has been successfully implemented and utilized in the clinical chemistry and hematology laboratories (1, 2). In microbiology, where rapid turnaround times are critical for the treatment of life-threatening infections, partial automation has been successfully implemented for blood culture and mycobacterial culture systems (3). New technological advances in automation and a projected shortage of skilled laboratory technologists have further prompted the development of solutions for microbiology laboratory automation (1).

Total laboratory automation generally refers to an integrated model of automation joined by a conveyor system (4). In microbiology, this includes automated processing of specimens, incubation, imaging of plates, reading of high-resolution plate images, discarding of plates when results are final, and delivery of plates to workbenches. The microbiology laboratory automation systems currently available include Copan WASPLab (Copan Diagnostics, Italy) and BD Kiestra (BD Kiestra B.V., Drachten, The Netherlands) (5, 6).

Matrix-assisted laser desorption ionization–time of flight mass spectrometry (MALDI-TOF MS) has revolutionized organism identification in the clinical microbiology laboratory (7). MALDI-TOF MS utilizes the principles of mass spectrometry for analysis of bacterial proteins to detect a unique signature that is compared to a curated database for identification (8). Compared to biochemical methods of identification that require further bacterial growth and incubation, MALDI-TOF MS is able to identify bacterial species more quickly and accurately and at a lower cost (3, 7, 9, 10).

It appears logical that total laboratory automation would be able to reduce turnaround time (TAT) and increase efficiency in the microbiology laboratory. Mutters et al. (11) demonstrated a reduction in turnaround times with implementation of both total laboratory automation and MALDI-TOF MS for organisms recovered from blood samples, while Graham et al. (12) demonstrated improved standardization and shortened the time to first plate reading in a prospective study comparing conventional processing to TLA. Total laboratory automation in clinical chemistry has been shown to decrease turnaround times and affect length of hospital stay (13, 14).

Our laboratory implemented MALDI-TOF MS (Bruker Daltonics, Billerica, MA) for organism identification (ID) in October 2013. Subsequently, we were the first clinical microbiology laboratory in the United States to implement the BD Kiestra TLA platform, in December 2014. Urine specimens were the first to be automated. The aim of this study was to assess the impact of MALDI-TOF MS and TLA, used both individually and together, on urine culture report turnaround times.

## MATERIALS AND METHODS

This study was performed in a four-hospital health care system with a total of 850 beds and a network of 2,000 physicians. The clinical microbiology laboratory performs approximately 300,000 billable tests per year, including 60,000 urine cultures.

### Conventional processing and identification.

Inpatient and outpatient urine specimens arrived for processing throughout the day. For the conventional plating technique, urine specimens were manually streaked using calibrated loops onto MacConkey and blood agar plates and manually placed into incubators. Specimen plating and incubation occurred 24 h per day. Plates were incubated overnight and read in two batches during the day shift (6 a.m. to 5 p.m.). A minimum of 14 h of incubation was required before reading and interpretation. Most plates were read in batch 1 starting at 7 a.m. The second batch, including those completing 14 h of incubation after 7 a.m., were read at 1 p.m. Spot tests, including indole, pyrrolidonyl arylamidase (PYR), leucine aminopeptidase (LAP), urease, oxidase, catalase, coagulase, and latex agglutination tests for Staphylococcus aureus and beta-hemolytic streptococci, were performed as appropriate for identification. Isolates not identified using spot testing were identified using the Vitek GNI or API20E kit (bioMérieux, Marcy-l'Étoile, France). Antimicrobial susceptibility testing (AST) of Gram-negative rods was performed by disk diffusion, while testing of Gram-positive cocci was performed by disk diffusion and overnight broth microdilution. In our laboratory information system (Soft Computer Corporation [SCC], Clearwater, FL), an interim report represents the time to an isolate identification result, a preliminary report represents the time to a preliminary negative result, and a final report for a positive specimen represents the time to an isolate AST result.

### Conventional processing and MALDI-TOF MS identification.

Bruker Biotyper MALDI-TOF MS was implemented in October 2013. It replaced identification of all isolates by the conventional methods listed in the above paragraph. Times to results for positive isolates, preliminary negative results, and antimicrobial testing reports were as described above.

### Kiestra TLA and MALDI-TOF MS identification.

BD Kiestra TLA was implemented in December 2014. Using TLA, urine was inoculated onto MacConkey and blood agar plates by the fully automated InoqulA system, and plates were transported via conveyor tracks (ProceedA) to the incubators (ReadA Compact). Digital images of the plates were taken at user-specified times (12 h and 30 h for urine cultures) and were interpreted by technologists throughout the day shift (6 a.m. to 5 p.m.) as images became available. Positive plates were delivered to the workbench (ReadABrowser), where a technologist performed MALDI-TOF MS for identification and prepared a 0.5 McFarland suspension for AST, when required. No spot or conventional identification procedures were used during the MALDI (conventional plating with MALDI-TOF MS identification) or TLA periods. Plates were interpreted using the same algorithm to select potential pathogens for identification and AST and to identify cultures as negative or not necessary to work up as during the conventional period. All plates interpreted as no growth or no pathogens at 12 h were incubated for an additional 18 h (total of 30 h) before a second image and interpretation occurred. Times to results for positive isolates, preliminary negative results, and antimicrobial testing reports were as described above.

### Data extraction and analysis.

To evaluate the impact of TLA on overall turnaround time (TAT) (comparison I), laboratory data for all urine cultures was extracted from our laboratory information system (SCC) in two 6-month periods corresponding to pre-Kiestra TLA implementation (January to June 2014) and post-Kiestra TLA implementation (January to June 2015). During both time periods, MALDI-TOF MS was used for identification of isolates. Turnaround times to identification, preliminary negative results, and AST reports for all urine cultures were measured from time of plate inoculation to entry of result into the electronic medical record.

To evaluate the impact of MALDI-TOF MS and TLA, both individually and together (comparison II), TAT to organism ID and AST reports from positive urine cultures were extracted in 1-month periods corresponding to (i) conventional plating and biochemical identification (CONV [October 2013]), (ii) conventional plating with MALDI-TOF MS identification (MALDI [March 2014]), (iii) early implementation of TLA with MALDI-TOF identification (TLA1 [April 2015]), and (iv) late implementation of TLA with MALDI-TOF identification (TLA2 [October 2015]). There was no difference in workflow between TLA1 and TLA2. The difference was the level of staff familiarity with the TLA system.

The following exclusion criteria were applied to all results: cultures with multiple reportable organisms and TAT outliers. Outliers were defined as reports with TATs 4 standard deviations above the median or a TAT of less than 6 h. The excluded outliers were specimens that did not follow standard processing and identification pathways.

Turnaround times for comparisons I and II were analyzed using Mann-Whitney U test and Kruskal-Wallis and Dunn's multiple comparison tests, where applicable. All analyses calculated are two tailed, and *P* values of ≤0.05 were considered significant.

## RESULTS

**FIG 1:**
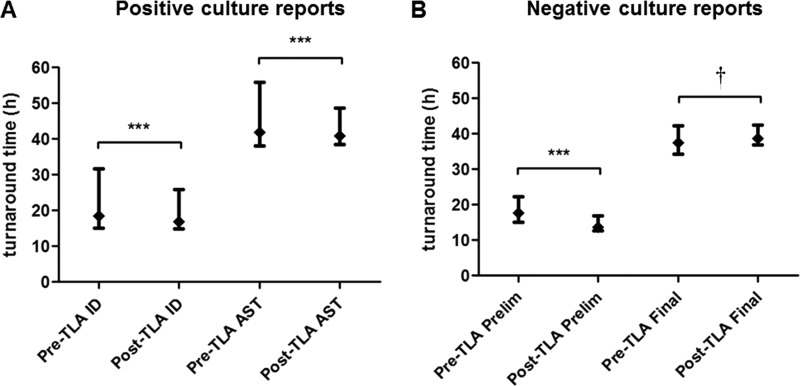
Comparison I: TATs (median and interquartile range) to AST, ID, and preliminary and final reports for (A) positive cultures (*n* = 17,251) and (B) negative cultures (*n* = 42,259) during the 6-month pre- and post-TLA periods. ***, *P* < 0.001; †, longer post-TLA final negative median TAT resulted from a change in reporting policy (see the text).

Data for comparison I from 61,157 positive and negative urine culture specimens were extracted, with 30,907 and 30,250 specimens from the pre- and post-TLA periods, respectively (Table 1). A total of 1,647 cultures were excluded from the analysis (823 and 824 from the pre- and post-TLA periods, respectively). The median TAT and interquartile range to organism identification for positive cultures decreased significantly from pre- to post-TLA (*P* < 0.001). For AST reports, the median TAT and interquartile ranges also showed significant decreases (*P* = 0.006) for positive cultures in the post-TLA period. TATs for preliminary negative results were significantly faster post-TLA (*P* < 0.001), but TATs to final negative reports did not improve (Table 2 and Fig. 1).

**TABLE 1:** Urine cultures from two 6-month periods pre- and post-total laboratory automation

Parameter	No. (%) of cultures^*a*^	
	Pre-TLA	Post-TLA
Total	30,907 (100)	30,250 (100)
1 organism reported	9,177 (29.7)	8,074 (26.7)
Multiple organisms reported	713 (2.3)	718 (2.4)
No pathogens reported	20,907 (67.6)	21,352 (70.6)
Outliers	110 (0.4)	106 (0.4)

a A decline in the number of reported positive cultures was observed (predominantly in cultures with urogenital flora, while a corresponding increase in reports with mixed bacterial flora was noted). The rate of detection for uropathogens remained constant.

**TABLE 2:** Comparison I: median turnaround time before and after implementation of total laboratory automation

Culture type (*n*)	Time (h) to^*a*^:			
	ID or preliminary negative result		AST or final negative result	
	Pre-TLA	Post-TLA	Pre-TLA	Post-TLA
Negative (42,259)	17.73 (14.97–22.25)	13.62 (12.60–16.80)***	37.38 (34.35–42.17)†	38.62 (36.85–42.53)†
Positive (17,251)	18.53 (15.00–31.62)	16.92 (14.95–25.87)***	41.80 (38.08–55.78)	40.85 (38.53–8.68)***

a Results are shown as the median turnaround time in hours (with interquartile range in parentheses) before and after implementation of total laboratory automation (TLA). ***, *P* < 0.001 post-TLA compared to pre-TLA; †, longer post-TLA final negative median TAT resulted from a change in reporting policy (see the text).

**FIG 2:**
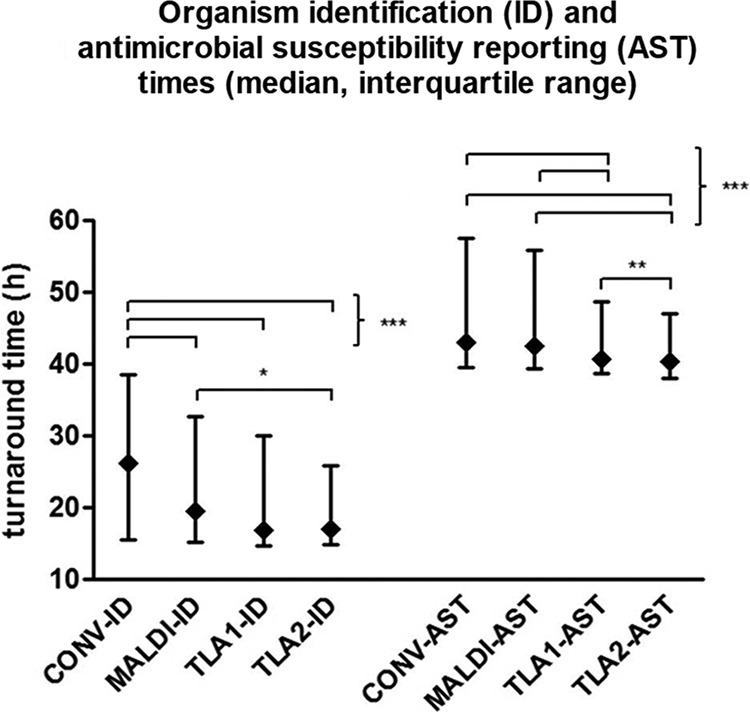
Comparison II: TATs (median and interquartile range) to ID and AST reporting during 1-month periods for conventional (CONV; *n* = 1,532), manual plating and MALDI-TOF MS (MALDI; *n* = 1,330), early total laboratory automation (TLA1; *n* = 1,214), and late total laboratory automation (TLA2; *n* = 1,326). *, *P* < 0.05; **, *P* < 0.01; ***, *P* < 0.001.

Comparison II analysis of the individual and combined effects of MALDI-TOF MS and TLA yielded 1,532, 1,330, 1,214, and 1,326 positive urine cultures for the CONV, MALDI, TLA1, and TLA2 periods, respectively. The median TAT to organism ID reports was significantly reduced with the introduction of MALDI-TOF MS (21.33 h for CONV and 18.02 h for MALDI) and was further reduced with the subsequent introduction of TLA (16.45 h for TLA1 and 16.79 h for TLA2 compared to 18.02 h for MALDI). The reduction in TATs with TLA was also seen for AST reports, which were significantly reduced from 42.74 h (CONV) and 42.33 h (MALDI) to 40.70 h and 40.33 h (TLA1 and TLA2, respectively) (Table 3 and Fig. 2). Both early (TLA1) and late (TLA2) TLA demonstrated a significant reduction in TAT variability compared to CONV and MALDI. Late implementation of TLA (TLA2) also demonstrated a reduction in TAT variability compared to early implementation (TLA1) for AST results only (Table 3 and Fig. 2).

**TABLE 3:** Comparison II: median turnaround time for the conventional, MALDI-TOF MS, and early and late total laboratory automation

Parameter	Result (h) for^*a*^:			
	CONV	MALDI	TLA1	TLA2
Overall:				
Time to ID	21.33 (14.96–36.79)	18.02 (14.70–30.70)	16.45 (14.67–24.28)	16.79 (14.83–23.55)
Time to AST	42.74 (39.55–57.43)	42.33 (39.30–55.38)	40.70 (38.65–47.73)	40.33 (38.07–47.08)
Spot test organisms				
Time to ID	17.11 (14.30–31.52)	17.60 (14.45–29.27)	16.30 (14.57–22.91)	16.45 (14.78–22.50)
Time to AST	42.49 (39.42–57.20)	42.18 (39.20–55.25)	40.53 (38.48–46.97)	40.02 (37.95–46.17)
Non-spot test organisms				
Time to ID	43.29 (39.40–58.13)	21.07 (16.87–33.89)	18.08 (15.42–37.34)	17.80 (15.12–26.73)
Time to AST	43.40 (40.09–58.66)	43.17 (40.40–56.31)	41.58 (39.23–50.16)	40.73 (38.27–50.74)

a Results are shown as the median turnaround time in hours (with interquartile range in parentheses) for the conventional (CONV), manual plating and MALDI-TOF MS (MALDI), and early (TLA1) and late (TLA2) total laboratory automation periods.

**FIG 3:**
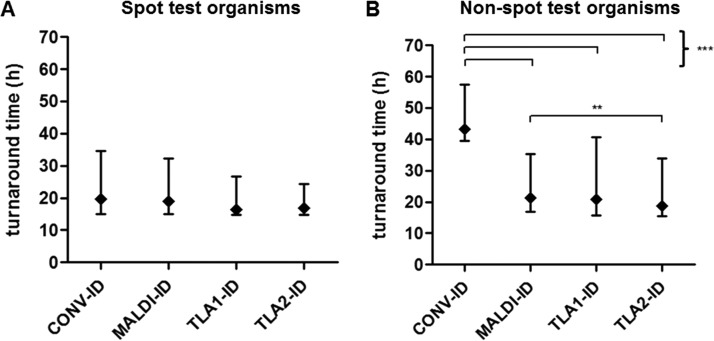
Effect of MALDI-TOF on turnaround times (median and interquartile range) for organism ID stratified by (A) spot test (*n* = 4,279) or (B) non-spot test (*n* = 1,123) methods prior to the introduction of MALDI-TOF. **, *P* < 0.01; ***, *P* < 0.001.

Organism identification during the conventional time period was analyzed according to the use of rapid biochemical spot tests versus Vitek GNI or API20E kit identifications requiring incubation. The use of MALDI-TOF MS or TLA had no effect on time to organism identification compared with rapid biochemical spot tests (17.11 h for CONV, 17.60 h for MALDI, 16.30 h for TLA1, and 16.45 h for TLA2); however, for organisms identified using Vitek GNI or API20E kits, MALDI-TOF MS significantly reduced the time to organism ID reports from 43.29 h (CONV) to 21.07 h (MALDI) (Fig. 3). Times to AST reports were significantly reduced after implementation of TLA. For both groups of organisms, a similar TAT-to-AST reduction trend was seen (Table 3).

## DISCUSSION

Comparison I of two 6-month time periods that included over 61,000 total specimens before and after implementation of TLA showed significantly improved TAT and TAT variability for positive reports, including identifications and results of antimicrobial testing (Table 2 and Fig. 1). This is in agreement with previously published side-by-side, but limited, comparisons of TLA versus conventional handling of positive blood and urine cultures (11, 12); our review provided exact times of computer reporting in the patient electronic medical record for antimicrobial reports and negative reports, as well as reports of organism identification. Although we did not quantitate data in a way that specifically identified the reason for TAT improvement, reduced TATs are likely due to automated plate inoculations that result in more isolated colonies and less need to subculture, elimination of temperature fluctuation during automated incubation and plate removal from incubators for examination, and the immediate availability of imaged plates after 12 h of incubation for identification and antimicrobial testing compared to batch reading of plates that were manually inoculated followed by conventional incubation (11–16). A separate but related analysis of our urine culture data during both time periods also demonstrated an increase in the number of plates with mixed bacterial flora, but a consistent detection rate for uropathogens after the introduction of TLA (data not shown).

Comparison I also demonstrated significant improvement in preliminary negative result TATs using TLA (Table 2 and Fig. 1B). This is a reflection of negative culture reports after the first 12-h incubation period (Fig. 1B). Improved incubation conditions, as mentioned above, allow colony recognition as early as 12 h postinoculation. Approximately 70% of potential pathogens detected were reported after 12 h of incubation. The remainder that required longer incubation included slower-growing organisms such as Candida species, Aerococcus species, and Actinotignum species (data not shown). This improved workflow provides more rapid reporting of positive results and more consistent TAT for both negative and positive results. Final negative-result TATs, those extending through the second incubation period that includes an additional 18 h, were not reduced by the use of TLA (Table 2 and Fig. 1B). This can be explained by a change in reporting and incubation policies for negative cultures. Before the introduction of TLA, negative urine cultures from specimens with a negative leukocyte esterase test were reported as final after 14 h of incubation; with TLA, all negative cultures are reported as final after a full 30 h of incubation.

Comparison II described a separate analysis of the impact of MALDI-TOF MS identification with and without TLA processing compared to conventional identification and processing. When MALDI-TOF MS for identification replaced conventional identification methods, significant decreases in median time and variability in time to identification resulted (Table 3 and Fig. 2). When organism identification data were examined closely, the use of MALDI-TOF MS reduced the time to identification for bacteria requiring identification systems, such as Vitek in our laboratory but was comparable to bacterial identification using spot testing (Table 3 and Fig. 3A and B). This is expected since biochemical spot tests are designed for simple and rapid organism ID, whereas identification systems require incubation time before providing a result. Even though MALDI-TOF MS was comparable in speed to spot testing, the dramatic improvement in MALDI-TOF MS organism ID TAT compared to non-spot testing lowered the overall TAT significantly. When TLA processing was added to MALDI-TOF MS for identification, a significant reduction in median time and variability to identification and antimicrobial reports compared to MALDI-TOF MS with conventional processing again resulted (Table 3 and Fig. 2). This is an important finding: TLA when combined with MALDI-TOF MS for organism identification provides additional reduction in TATs compared to MALDI-TOF MS without TLA. As one would expect, the additive effect of both MALDI-TOF MS plus TLA processing significantly reduced median TATs and TAT variability for identifications and antimicrobial reports compared to conventional methods (Table 3 and Fig. 2). It is noted that the significant differences in median TATs are small and may not be clinically important, however, the differences in interquartile ranges are likely to be impactful. For instance, more results fell outside of a 48-h window during the pre-TLA period (33.7%) compared with post-TLA (26.2%, *P* < 0.001); thus, for a number of cases, the turnaround time improvement can be significant.

Although implementation of MALDI-TOF MS and TLA was associated with decreased TAT, clinical and economic effects were not investigated in this study. Implementation of TLA in our laboratory did result in a 20% reduction in full-time-equivalents (FTEs), and in a separate evaluation, Shibib et al. showed that TLA allowed technologists to perform 25% more tests per FTE (17). Others have shown that rapid identification of organisms in blood cultures resulting in reduced TAT to organism identification was associated with decreased intensive care unit (ICU) length of stay and 30-day mortality (18). We suggest that reduced TATs for all microbiology reports have potential for positive clinical impact.

Limitations of this study include retrospective data collection and comparison of sequential time periods. In an effort to address these concerns, extended time periods (6 months) and data for large numbers of specimens (>61,000 urines) were gathered for comparison I. Comparison II used four time periods of 1-month duration each with over 1,200 positive specimens in each period. An additional limitation is the accuracy of the laboratory information system (SCC) data representing the time of identification, antimicrobial test, and preliminary reports. Time to identification of isolates when multiple isolates were reported could not be determined for each isolate. The proportion of urine cultures with multiple pathogens reported was small and unlikely to have a significant impact on overall TATs. The pre-TLA (30,907 specimens) and post-TLA (30,250 specimens) periods in comparison I contained only 2.3% and 2.4% of specimens with two pathogens reported, respectively (Table 1). The protocol for three or more pathogens present requires reporting as grossly contaminated with no identification or AST results. Therefore, only specimens with one pathogen were included. The time to the antimicrobial testing report was represented by the final report since antimicrobial results are the last data added. Rare final reports were delayed because of mixed cultures, add-on antimicrobials to be tested by the clinical service, and technical problems requiring repeat testing. The greatest of these were removed by eliminating outliers defined as TATs 4 standard deviations above the median. These outliers formed a small proportion of the overall data, and approximately equal numbers were removed from each group. Finally, new technology requires training and acclimation. During the break-in period, facility with the system may be decreased. To avoid a bias against technology, we evaluated data from early and late TLA adoption. A small but significant reduction in TAT was seen with AST reports; however, time to identification was not changed (Table 3 and Fig. 2).

In conclusion, implementation of MALDI-TOF MS and Kiestra TLA individually and when used together significantly decreased turnaround time and turnaround time variability for organism identifications, AST reports, and negative urine culture reports. More rapid reporting by other technologies, such as detection of bacterial species and resistant markers in positive blood cultures by molecular probe and nucleic acid amplification do result in improved patient care (18). It will be important to connect more rapid identification and antimicrobial reports resulting from TLA with patient outcomes.
